# Supplementary material for: Theory predicts UV/vis-to-IR photonic down conversion mediated by excited state vibrational polaritons
Source: Nat Commun. 2023 Aug 9;14:4804. doi: 10.1038/s41467-023-40400-z (PMC10412565; doi:10.1038/s41467-023-40400-z)
Supplement: Supplementary file 1 — Supplementary Information [file 41467_2023_40400_MOESM1_ESM.pdf]

Supplementary Information for UV/vis-to-IR Photonic Down Conversion Mediated by Excited State Vibrational Polaritons

Connor K. Terry Weatherly, Justin Provazza, Emily A. Weiss,* and Roel Tempelaar*

Department of Chemistry, Northwestern University, 2145 Sheridan Rd., Evanston, IL 60208-3113

E-mail: e-weiss@northwestern.edu; roel.tempelaar@northwestern.edu

Contents

Supplementary Note 1. Pauli-Fierz Hamiltonian Details **S2**

Full Expansion of the Vibrational Polariton Pauli-Fierz Hamiltonian S2

Vibrational Polariton Potential Energy Surface and Hessian Matrix S5

Supplementary Note 2. Derivation of the Analytic $\langle \hat{N}_{\mathbf{cav}}(t) \rangle$ Expression for the Isolated Cavity-Vibration System **S6**

Classical Polariton Trajectories S6

Vibrational Polariton Wigner Function S8

Weyl Function of the Cavity Photon Number Operator S9

$\langle \hat{N}_{\text{cav}}(t) \rangle$ Expression S10

Supplementary Note 3. Cavity Polarization Due to the Molecular Dipole **S11**

Supplementary Note 4. Effect of the IR Dipole Self Energy **S13**

Supplementary Note 5. Isolated Cavity-Vibration System at Non-resonant Conditions **S13**

Supplementary Note 6. Dissipative Cavity-Vibration System at Non-resonant Conditions **S17**

Supplementary Note 7. Effect of the Non-Radiative Lifetime on % Emission for the Open Cavity-Vibration System **S18**

Supplementary Note 8. Absorption Response Function for Pyrene **S19**

Supplementary references **S19**

Supplementary Note 1. Pauli-Fierz Hamiltonian Details

This section provides an in depth analysis of the vibrational polariton Pauli-Fierz model Hamiltonian. As is done in the main text, we express the Hamiltonian with a linear expansion of the dipole operator. However, here we briefly discuss all terms that are contained within the Hamiltonian, where in the maintext we ignored all interaction terms except for the bilinear coupling between the cavity and the vibration.

Full Expansion of the Vibrational Polariton Pauli-Fierz Hamiltonian

We consider a electronic two-level system, containing a single vibrational mode, embedded in a Fabry-Perot cavity tuned so that its fundamental frequency, ω_c, is in the infrared range. The vibronic coupling within the molecule is captured by a shift in the excited state potential energy surface (PES), where the ground state nuclear equilibrium position ($Q_0^{(g)}$) is chosen to

be at the origin of the nuclear coordinate such that $Q_0^{(g)} = 0$. The Pauli-Fierz Hamiltonian is expanded in the molecular diabatic basis containing a ground and excited electronic basis state, $|g\rangle$ and $|e\rangle$, respectively, which are given by Eq. (2) of the maintext. Accordingly, the Pauli-Fierz Hamiltonian in this basis is

$$\hat{H} = \left[\frac{\hat{P}^2}{2} + \frac{\hat{p}_c^2}{2} + \frac{1}{2}\omega_v^2\hat{Q}^2 + \frac{1}{2}\omega_c^2\hat{q}_c^2\right]\hat{\mathbb{1}} + (E_{\text{vert}} - \omega_v^2 Q_0^{(e)}\hat{Q})\,|e\rangle\langle e| \\ + \frac{1}{2}\omega_c^2 \sum_{\alpha\beta\in\{g,e\}} \left[2\boldsymbol{A}\cdot\boldsymbol{\mu_{\alpha\beta}}\,\hat{q}_c\,\hat{Q} + (\boldsymbol{A}\cdot\boldsymbol{\mu_{\alpha\beta}})^2\right]|\alpha\rangle\langle\beta|\,, \tag{S1}$$

where $\hat{p}_c$ and $\hat{q}_c$ are momentum and position operators, respectively, of the cavity mode with frequency ω_c, $\boldsymbol{A} = \sqrt{2/\hbar\omega_c}A_0\boldsymbol{\epsilon_c}$ with $A_0 = \sqrt{\hbar/2\omega_c\varepsilon V}$ being the amplitude of the cavity mode vector potential, ε being the effective permittivity of the cavity, V being the cavity mode volume, and $\boldsymbol{\epsilon_c}$ being the cavity mode polarization unit vector. Also, $\hat{P}$ and $\hat{Q}$ are the mass weighted momentum and position operators, respectively, of the molecular vibrational mode with frequency ω_v, $E_{\text{vert}} = \hbar\Omega_{e,g} + S\hbar\omega_v$ is the energy of the vertical transition, $Q_0^{(e)}$ is the excited state nuclear equilibrium configuration, $\boldsymbol{\mu_{\alpha\beta}} = \langle\alpha|\,\hat{\boldsymbol{\mu}}\,|\beta\rangle$ is $\alpha\beta$ element of the dipole operator, and $\hat{\mathbb{1}} = |e\rangle\langle e| + |g\rangle\langle g|$. We assume $\boldsymbol{\mu_{\alpha\beta}} = 0$ when $\alpha \neq \beta$ and expand $\boldsymbol{\mu_{\alpha\alpha}}$ to the first order about the ground state equilibrium configuration to account for linear coupling of the vibration to the cavity,

$$\boldsymbol{\mu_{\alpha\alpha}}(Q) = \boldsymbol{\mu^0_\alpha} + \boldsymbol{\mu'_\alpha}\hat{Q}, \tag{S2}$$

where

$$\boldsymbol{\mu^0_\alpha} \equiv \boldsymbol{\mu_{\alpha\alpha}}(Q_0^{(g)}) - \boldsymbol{\mu'_\alpha}Q_0^{(g)}, \tag{S3}$$

and where

$$\boldsymbol{\mu'_\alpha} \equiv \left.\frac{\partial\boldsymbol{\mu_{\alpha\alpha}}(Q)}{\partial Q}\right|_{Q_0^{(g)}}. \tag{S4}$$

Substituting Eq. (S2) into Eq. (S1) and expanding the result gives the following terms,

$$\hat{H} = \hat{H}_0 + \hat{H}_{\text{el-vib}} + \hat{H}_{\text{PFS}} + \hat{H}_{\text{cav-vib}} + \hat{H}_{\text{dipole}} + \hat{H}_{\text{NMES}} + \hat{H}_{\text{DR}}. \tag{S5}$$

The different terms of Eq. (S5) are as follows:

$$\hat{H}_0 = \left[\frac{\hat{P}^2}{2} + \frac{\hat{p}_c^2}{2} + \frac{1}{2}\omega_v^2\hat{Q}^2 + \frac{1}{2}\omega_c^2\hat{q}_c^2\right]\hat{\mathbb{1}} \tag{S6}$$

describes the cavity field and molecular normal mode when they are uncoupled from each other.

$$\hat{H}_{\text{el-vib}} = (E_{\text{vert}} - \omega_v^2 Q_0^{(e)}\hat{Q})\,|e\rangle\,\langle e| \tag{S7}$$

describes the shift (due to vibronic coupling) and energetic difference of the molecule's excited and ground state PES.

$$\hat{H}_{\text{PFS}} = \sum_{\alpha\in\{g,e\}}\left[\omega_c^2\boldsymbol{A}\cdot\boldsymbol{\mu}^{\mathbf{0}}_{\boldsymbol{\alpha}}\hat{q}_c\right]|\alpha\rangle\,\langle\alpha| \tag{S8}$$

describes the cavity field equilibrium shift, which defines a "polarized Fock state" (PFS).[1]

$$\hat{H}_{\text{cav-vib}} = \sum_{\alpha\in\{g,e\}}\left[\omega_c^2\boldsymbol{A}\cdot\boldsymbol{\mu}'_{\boldsymbol{\alpha}}\hat{Q}\,\hat{q}_c\right]|\alpha\rangle\,\langle\alpha| \tag{S9}$$

is the cavity-vibration bilinear coupling that mixes the cavity mode with the vibration. It is the only cavity-vibration interaction term used in the main text. Note that the coupling is different on the ground and excited state if $\boldsymbol{\mu}'_{\boldsymbol{e}} \neq \boldsymbol{\mu}'_{\boldsymbol{g}}$.

$$\hat{H}_{\text{dipole}} = \sum_{\alpha\in\{g,e\}}\left[\frac{1}{2}(\omega_c^2\boldsymbol{A}\cdot\boldsymbol{\mu}^{\mathbf{0}}_{\boldsymbol{\alpha}})^2\right]|\alpha\rangle\,\langle\alpha| \tag{S10}$$

is the reference configuration-dependent dipole self energy due to the cavity field. If $\boldsymbol{\mu}^{\mathbf{0}}_{\boldsymbol{g}} \neq \boldsymbol{\mu}^{\mathbf{0}}_{\boldsymbol{e}}$ then the molecule's vertical excitation energy will change in the presence of a cavity, relative

to the molecule outside of the cavity.

$$\hat{H}_{\text{NMES}} = \sum_{\alpha \in \{g,e\}} \left[\omega_c^2 (\boldsymbol{A} \cdot \boldsymbol{\mu}_{\boldsymbol{\alpha}}^{\boldsymbol{0}}) \, (\boldsymbol{A} \cdot \boldsymbol{\mu}'_{\boldsymbol{\alpha}}) \, \hat{Q} \right] |\alpha\rangle \langle\alpha| \tag{S11}$$

describes a molecular normal mode equilibrium shift (NMES) due to the reference configuration permanent dipole interacting with the vibrational transition dipole through the cavity field.

$$\hat{H}_{\text{DR}} = \sum_{\alpha \in \{g,e\}} \left[\frac{1}{2} \left(\omega_c \boldsymbol{A} \cdot \boldsymbol{\mu}'_{\boldsymbol{\alpha}} \hat{Q} \right)^2 \right] |\alpha\rangle \langle\alpha| \tag{S12}$$

describes a cavity-induced molecular normal mode Duschinsky rotation (DR), which would differ in the ground and excited state if $\boldsymbol{\mu}'_{\boldsymbol{g}} \neq \boldsymbol{\mu}'_{\boldsymbol{e}}$.

Vibrational Polariton Potential Energy Surface and Hessian Matrix

We treat the cavity-vibration system as a two-dimensional harmonic PES (h_α) such that the vibrational polariton Hamiltonian can be written as

$$\hat{H} = \frac{\boldsymbol{p} \cdot \boldsymbol{p}}{2} + \sum_{\alpha} h_\alpha(\boldsymbol{q}) \, |\alpha\rangle \langle\alpha| \, , \tag{S13}$$

where $\boldsymbol{q} = \left(\begin{smallmatrix} \hat{q}_c \\ \hat{Q} \end{smallmatrix}\right)$ and $\boldsymbol{p} = \left(\begin{smallmatrix} \hat{p}_c \\ \hat{P} \end{smallmatrix}\right)$ are position and momentum vectors in the cavity-vibration basis, respectively. h_α is then expanded in a Taylor series about the reference configuration, $\boldsymbol{q_0} = \left(\begin{smallmatrix} 0 \\ Q_0^{(g)} \end{smallmatrix}\right)$,

$$\hat{H} = \frac{\boldsymbol{p} \cdot \boldsymbol{p}}{2} + \sum_{\alpha} \left[h_\alpha(\boldsymbol{q_0}) + \nabla h_\alpha(\boldsymbol{q_0})(\boldsymbol{q} - \boldsymbol{q_0}) + \frac{1}{2}(\boldsymbol{q} - \boldsymbol{q_0})^T \boldsymbol{H}^\alpha (\boldsymbol{q} - \boldsymbol{q_0}) \right] |\alpha\rangle \langle\alpha| \, . \tag{S14}$$

Here, $\boldsymbol{H}^\alpha$ is the Hessian matrix with elements defined by $\boldsymbol{H}_{ij}^\alpha = \frac{\partial^2 h_\alpha}{\partial q_i \partial q_j}\Big|_{\boldsymbol{q_0}}$ where $q_i, q_j = q$ or Q. The cavity-vibration PES, h_α, is determined such that Eq. (S14) becomes Eq. (S5).

Therefore, the vibrational polariton Hessian Matrix for the α electronic state becomes

$$\boldsymbol{H}^{\alpha} = \begin{pmatrix} \omega_c^2 & G_\alpha \\ G_\alpha^* & \omega_v^2 + (\omega_c \boldsymbol{A} \cdot \boldsymbol{\mu}'_{\boldsymbol{\alpha}})^2 \end{pmatrix}, \tag{S15}$$

with

$$G_\alpha = A_0 \boldsymbol{\epsilon_c} \cdot \boldsymbol{\mu}'_{\boldsymbol{\alpha}} \sqrt{\frac{2\omega_c^3}{\hbar}} \tag{S16}$$

being the bilinear coupling constant. By transforming into a basis such that $\boldsymbol{H}^{\alpha}$ is diagonal, Eq. (S14) describes a system of uncoupled harmonic oscillators. This basis is the polariton normal mode basis and is found by determining the eigenvalues and eigenvectors of $\boldsymbol{H}^{\alpha}$.

Supplementary Note 2. Derivation of the Analytic $\langle \hat{N}_{\mathbf{cav}}(t) \rangle$ Expression for the Isolated Cavity-Vibration System

Here we show the derivation of the time-dependent expectation value of the cavity mode occupation number, $\hat{N}_{\text{cav}}(t)$, at finite temperature, for when the cavity vibration system is not coupled to any baths.

Classical Polariton Trajectories

As is done in the main text, we take all cavity-vibration interaction terms to be zero except for $\hat{H}_{\text{cav-vib}}$ such that

$$\hat{H} = \hat{H}_0 + \hat{H}_{\text{el-vib}} + \hat{H}_{\text{cav-vib}}. \tag{S17}$$

The electronic excited state term of the Pauli-Fierz Hamiltonian describing the cavity-vibration system can be written in vector notation as

$$H^e = \frac{\boldsymbol{p} \cdot \boldsymbol{p}}{2} + \frac{\boldsymbol{q}^T \boldsymbol{H}^e \boldsymbol{q}}{2} + E_{\text{vert}} - \omega_v^2 Q_0^{(e)} \boldsymbol{q} \cdot \boldsymbol{\epsilon}_v, \tag{S18}$$

where $\boldsymbol{\epsilon}_v = \binom{0}{1}$ is the vibrational mode unit vector and

$$\boldsymbol{H}^e = \begin{pmatrix} \omega_c^2 & G_e \\ G_e^* & \omega_v^2 \end{pmatrix} \tag{S19}$$

is the hessian matrix. Let $\boldsymbol{U}$ be the unitary matrix such that

$$\boldsymbol{U}^T \boldsymbol{H}^e \boldsymbol{U} = \boldsymbol{D} = \begin{pmatrix} \Omega_+^2 & 0 \\ 0 & \Omega_-^2 \end{pmatrix}, \tag{S20}$$

where $\boldsymbol{D}$ is the diagonal matrix containing the eigenvalues of $\boldsymbol{H}^e$ on its diagonal elements. Furthermore,

$$\boldsymbol{U} = \begin{pmatrix} c_c^{(+)} & c_v^{(+)} \\ c_c^{(-)} & c_v^{(-)} \end{pmatrix}, \tag{S21}$$

where $c_\alpha^{(\gamma)}$ is the projection of the vibration or cavity unit vector (corresponding to $\alpha = v$ or c) onto the upper or lower polariton unit vector (corresponding to $\gamma = +$ or $-$), and whose columns make up the eigenvectors of $\boldsymbol{H}^e$.

Using the property $\boldsymbol{U}\boldsymbol{U}^T = \mathbb{1}$, the Hamiltonian in Eq. (S18) can be expanded in the polariton basis,

$$\begin{aligned} \hat{H}^e &= \frac{\boldsymbol{p}^T \boldsymbol{U}\boldsymbol{U}^T \boldsymbol{p}}{2} + \frac{\boldsymbol{q}^T \boldsymbol{U}\boldsymbol{U}^T \boldsymbol{H}^e \boldsymbol{U}\boldsymbol{U}^T \boldsymbol{q}}{2} + E_{\text{vert}} - \omega_v^2 Q_0^{(e)} \boldsymbol{q}\boldsymbol{U}\boldsymbol{U}^T \boldsymbol{\epsilon}_v \\ &= \frac{\boldsymbol{p}_p \cdot \boldsymbol{p}_p}{2} + \frac{\boldsymbol{r}_p^T \boldsymbol{D} \boldsymbol{r}_p}{2} + E_{\text{vert}} - \omega_v^2 Q_0^{(e)} \boldsymbol{r}_{\boldsymbol{p}} \cdot \binom{c_v^{(+)}}{c_v^{(-)}} \\ &= E_{\text{vert}} + \sum_{\gamma=+/-} \frac{\hat{P}_\gamma^2}{2} + \frac{\Omega_\gamma^2 \hat{R}_\gamma^2}{2} - \omega_v^2 Q_0^{(e)} c_v^{(\gamma)} \hat{R}_\gamma, \end{aligned} \tag{S22}$$

where $\boldsymbol{U}^T \boldsymbol{q} = \boldsymbol{r}_p = \binom{\hat{R}_+}{\hat{R}_-}$ and $\boldsymbol{U}^T \boldsymbol{p} = \boldsymbol{p}_p = \binom{\hat{P}_+}{\hat{P}_-}$ are the polariton position and momentum vectors, respectively. Because Eq. (S22) does not have more than a quadratic dependence on the phase space operators, $\hat{R}_\gamma$ and $\hat{P}_\gamma$, within the truncated Wigner approximation (TWA) the dynamics of the system can be governed exactly by the Classical equations of motion

for a Hamiltonian given by the Weyl symbol of $\hat{H}^e$, which is found by simply replacing its phase space operators with classical position and momentum variables. The Weyl symbol of $\hat{H}^e$ (from Eq. (S22)) is the classical Hamiltonian for uncoupled harmonic oscillators that have been shifted by an amount, $\frac{\omega_v^2}{\Omega_\gamma^2} Q_0^{(e)} c_v^{(\gamma)}$. Hence, the classical trajectories describing the time evolution of the polaritons are given by

$$R_\gamma(t) = R_{0_\gamma} \cos(\Omega_\gamma t) + \frac{P_{0_\gamma}}{\Omega_\gamma} \sin(\Omega_\gamma t) + \frac{\omega_v^2}{\Omega_\gamma^2} c_v^{(\gamma)} Q_0^{(e)} (1 - \cos(\Omega_\gamma t)), \tag{S23}$$

with

$$\begin{aligned} P_\gamma(t) &= \dot{R}_\gamma(t) \\ &= P_{0_\gamma} \cos(\Omega_\gamma t) - \Omega_\gamma R_{0_\gamma} \sin(\Omega_\gamma t) + \frac{\omega_v^2}{\Omega_\gamma} c_v^{(\gamma)} Q_0^{(e)} \sin(\Omega_\gamma t), \end{aligned} \tag{S24}$$

where R_{0_γ} and P_{0_γ} are the initial position and momentum of the γ polariton mode.

Vibrational Polariton Wigner Function

The time-dependent cavity mode occupation number can be calculated according to

$$\langle \hat{N}_{\text{cav}}(t) \rangle = \int d\boldsymbol{r}_{\boldsymbol{p}}^{\boldsymbol{0}} \frac{d\boldsymbol{p}_{\boldsymbol{p}}^{\boldsymbol{0}}}{(2\pi\hbar)^2} N_{\text{cav}}^{(W)} \left(\boldsymbol{p}_{\boldsymbol{p}}(t), \boldsymbol{r}_{\boldsymbol{p}}(t)\right) W(\boldsymbol{p}_{\boldsymbol{p}}^{\boldsymbol{0}}, \boldsymbol{r}_{\boldsymbol{p}}^{\boldsymbol{0}}), \tag{S25}$$

where the $W(\boldsymbol{p}_{\boldsymbol{p}}^{\boldsymbol{0}}, \boldsymbol{r}_{\boldsymbol{p}}^{\boldsymbol{0}})$ is the Wigner function of the system at time zero before the vertical excitation has occurred and $N_{\text{cav}}^{(W)}$ is the Weyl symbol of $\hat{N}_{\text{cav}}(t)$. We choose $N_{\text{cav}}^{(W)}$ to carry the time dependence and W to be a distribution of the initial positions and momenta of the system, which simplifies our calculations compared to letting W carry the time dependence.

The Wigner function is found by taking the Weyl transform of the density matrix. In our case, at time zero, before the vertical excitation, the system is in a thermal distribution of electronic ground state vibrational polaritons. This gives rise to the following Wigner

distribution,

$$W(\boldsymbol{p_p^0}, \boldsymbol{r_p^0}) = \prod_{\gamma=+/-} \frac{\hbar}{\Omega_\gamma \sigma_{R_\gamma}^2} e^{-\frac{P_{0\gamma}^2}{2\Omega_\gamma^2 \sigma_{R_\gamma}^2} - \frac{R_{0\gamma}^2}{2\sigma_{R_\gamma}^2}}, \tag{S26}$$

with $\sigma_{R_\gamma} = \sqrt{\frac{\hbar}{2\Omega_\gamma \tanh(\beta\hbar\Omega_\gamma/2)}}$.

Weyl Function of the Cavity Photon Number Operator

The cavity photon operator is given by

$$\hat{N}_{\text{cav}} = \frac{1}{2\hbar\omega_c}(\omega_c^2 \hat{q}_c^2 + \hat{p}_c^2) - 1/2, \tag{S27}$$

where $\hat{q}_c$ and $\hat{p}_c$ are the cavity position and momentum operators, respectively. The Weyl symbol of $\hat{N}_{\text{cav}}$ is found by replacing the position and momentum operators by their respective phase space variables, i.e.,

$$N_{\text{cav}}^{(W)} = \frac{1}{2\hbar\omega_c}(\omega_c^2 q^2 + p^2) - 1/2, \tag{S28}$$

which can be written in vector notation by substituting $q = \boldsymbol{q} \cdot \boldsymbol{\epsilon_c}$ and $p = \boldsymbol{p} \cdot \boldsymbol{\epsilon_c}$, where $\boldsymbol{\epsilon}_c = \binom{1}{0}$ is the cavity mode unit vector. $N_{\text{cav}}^{(W)}$ can now be transformed into the polariton basis, giving,

$$N_{\text{cav}}^{(W)} = \frac{1}{2\hbar\omega_c}\left(\omega_c^2 \left(\sum_{\gamma=+/-} c_c^{(\gamma)} R_\gamma\right)^2 + \left(\sum_{\gamma=+/-} c_c^{(\gamma)} P_\gamma\right)^2\right) - 1/2. \tag{S29}$$

From the Gaussian form of the Wigner function in Eq. (S26), only terms containing even order dependence on the initial phase space variables will survive the integration in Eq. (S25). Therefore, when substituting Eq. (S23) and Eq. (S24) into Eq. (S29), we only

retain the following terms in the momentum and position expansions,

$$\left(\sum_\gamma c_c^{(\gamma)} P_\gamma(t)\right)^2 \to \sum_\gamma c_c^{(\gamma)2}\left(P_{0_\gamma}^2 \cos^2(\Omega_\gamma t) + \Omega_\gamma^2 R_{0_\gamma}^2 \sin^2(\Omega_\gamma t)\right) + \left(\sum_\gamma c_c^{(\gamma)} c_v^{(\gamma)\omega_v^2 Q_0^{(e)}} \frac{\sin(\Omega_\gamma t)}{\Omega_\gamma}\right)^2, \tag{S30}$$

and

$$\left(\sum_\gamma c_c^{(\gamma)} R_\gamma(t)\right)^2 \to \sum_\gamma c_c^{(\gamma)2}\left(R_{0_\gamma}^2 \cos^2(\Omega_\gamma t) + \frac{1}{\Omega_\gamma^2} P_{0_\gamma}^2 \sin^2(\Omega_\gamma t)\right) + 4\left(\sum_\gamma c_c^{(\gamma)} c_v^{(\gamma)\omega_v^2 Q_0^{(e)}} \frac{\sin^2(\Omega_\gamma t/2)}{\Omega_\gamma^2}\right)^2, \tag{S31}$$

where we have used that $\frac{1}{2}(1-\cos(x)) = \sin^2(x/2)$.

$\langle \hat{N}_{\mathbf{cav}}(t)\rangle$ Expression

Performing the integral in Eq. (S29) using the expressions for $N_{\text{cav}}^{(W)}$ and W, we obtain the time dependent expression for the expected number of cavity photons,

$$\langle \hat{N}_{\text{cav}}(t)\rangle = \frac{1}{2\hbar\omega_c}\left(\sum_\gamma c_c^{(\gamma)} c_v^{(\gamma)\omega_v^2 Q_0^{(e)}} \frac{\sin(\Omega_\gamma t)}{\Omega_\gamma}\right)^2 + \frac{2\omega_c}{\hbar}\left(\sum_\gamma c_c^{(\gamma)} c_v^{(\gamma)\omega_v^2 Q_0^{(e)}} \frac{\sin^2(\Omega_\gamma t/2)}{\Omega_\gamma^2}\right)^2 + N_\beta, \tag{S32}$$

with $N_\beta = \frac{1}{4}\sum_\gamma \left(c_c^{(\gamma)2}\left(\frac{\Omega_\gamma}{\omega_c} + \frac{\omega_c}{\Omega_\gamma}\right)\coth(\beta\hbar\Omega_\gamma/2)\right) - \frac{1}{2}$ describing the thermal contribution to the cavity mode number. The coeffecients, $c_c^{(\gamma)}$, and polariton frequencies, Ω_γ, are found by the expressions for the eigenvalues and eigenvectors of $\boldsymbol{H}^e$.

A similar procedure was done for the open polariton system, where the cavity was coupled to external electromagnetic field modes and the vibration was coupled to solvent phonon modes. In this case, the hessian matrix has dimensions of the DOF of the system and its eigenvalues and eigenvectors were calculated numerically.

Supplementary Note 3. Cavity Polarization Due to the Molecular Dipole

This sections discusses the effect of the cavity-vibration interactions terms, $\hat{H}_{\text{PFS}}$.

When $\sqrt{\frac{\hbar}{2\omega_c}}(\hat{a}+\hat{a}^\dagger)$ is substituted for $\hat{q}_c$, it is evident that the term

$$\hat{H}_{\text{PFS}} = \sum_{\alpha\in\{g,e\}} \left[\omega_c^2 \boldsymbol{A}\cdot\boldsymbol{\mu}_{\boldsymbol{\alpha}}^{\boldsymbol{0}}\hat{q}_c\right] |\alpha\rangle\langle\alpha| \tag{S33}$$

polarizes the cavity Fock states as this term mixes the bare cavity Fock states due to a shift in the cavity mode along q. This term is analogous to the mixing of ground state vibrational modes in the excited state due to vibronic coupling, but for the cavity Fock states.

The classical trajectories, when $\hat{H}_{\text{PFS}}$ is included, are

$$R^{\gamma_\alpha}(t) = R_0^{\gamma_\alpha}\cos(\Omega_{\gamma_\alpha}t) + \frac{P_0^{\gamma_\alpha}}{\Omega_{\gamma_\alpha}}\sin(\Omega_{\gamma_\alpha}t) + \frac{1}{\Omega_\gamma^2}\left(\omega_v^2 Q_0^{(\alpha)}c_v^{(\gamma_\alpha)} - \omega_c^2\boldsymbol{A}\cdot\boldsymbol{\mu}_{\boldsymbol{\alpha}}^{\boldsymbol{0}}c_c^{(\gamma_\alpha)}\right)(1-\cos(\Omega_{\gamma_\alpha}t)), \tag{S34}$$

with

$$\begin{aligned} P^{\gamma_\alpha}(t) &= \dot{R}^{\gamma_\alpha}(t) \\ &= P_0^{\gamma_\alpha}\cos(\Omega_{\gamma_\alpha}t) - \Omega_{\gamma_\alpha}R_0^{\gamma_\alpha}\sin(\Omega_{\gamma_\alpha}t) + \frac{1}{\Omega_\gamma}\left(\omega_v^2 Q_0^{(\alpha)}c_v^{(\gamma_\alpha)} - \omega_c^2\boldsymbol{A}\cdot\boldsymbol{\mu}_{\boldsymbol{\alpha}}^{\boldsymbol{0}}c_c^{(\gamma_\alpha)}\right)\sin(\Omega_{\gamma_\alpha}t). \end{aligned} \tag{S35}$$

Eqs. (S34) and (S35) indicate that the polariton harmonic oscillators have been shifted along their respective polariton modes by the amount $(\omega_v^2 Q_0^{(\alpha)}c_v^{(\gamma_\alpha)} - \omega_c^2\boldsymbol{A}\cdot\boldsymbol{\mu}_{\boldsymbol{\alpha}}^{\boldsymbol{0}}c_c^{(\gamma_\alpha)})/\Omega_\gamma^2$, where $-\omega_c^2\boldsymbol{A}\cdot\boldsymbol{\mu}_{\boldsymbol{\alpha}}^{\boldsymbol{0}}c_c^{(\gamma_\alpha)}/\Omega_\gamma^2$ comes from the inculsion of the $\hat{H}_{\text{PFS}}$ term in the Hamiltonian. It is therefore apparent that the polarization of the bare cavity Fock states by $\boldsymbol{\mu}_{\boldsymbol{\alpha}}^{\boldsymbol{0}}$ causes an equivalent effect as the vibronic coupling, which determines $Q_0^{(e)}$. The number of IR photons produced in the ESVP-mediated down conversion process would be increased when $\boldsymbol{A}\cdot\boldsymbol{\mu}_{\boldsymbol{g}}^{\boldsymbol{0}}c_c^{(\gamma_g)} < \boldsymbol{A}\cdot\boldsymbol{\mu}_{\boldsymbol{e}}^{\boldsymbol{0}}c_c^{(\gamma_e)}$ compared to when $\boldsymbol{A}\cdot\boldsymbol{\mu}_{\boldsymbol{g}}^{\boldsymbol{0}}c_c^{(\gamma_g)} = \boldsymbol{A}\cdot\boldsymbol{\mu}_{\boldsymbol{e}}^{\boldsymbol{0}}c_c^{(\gamma_e)}$ (which is the case when

$\hat{H}_{\mathrm{PFS}} = 0$). On the other hand, if $\boldsymbol{A} \cdot \boldsymbol{\mu}_{\boldsymbol{g}}^{\mathbf{0}} c_c^{(\gamma_g)} > \boldsymbol{A} \cdot \boldsymbol{\mu}_{\boldsymbol{e}}^{\mathbf{0}} c_c^{(\gamma_e)}$ then the number of IR photons would decrease compared to $\boldsymbol{A} \cdot \boldsymbol{\mu}_{\boldsymbol{g}}^{\mathbf{0}} c_c^{(\gamma_g)} = \boldsymbol{A} \cdot \boldsymbol{\mu}_{\boldsymbol{e}}^{\mathbf{0}} c_c^{(\gamma_e)}$. This result suggests that to maximize the ESVP-mediated down conversion process, the excited state permanent dipole at the FC point (more precisely, it's projection onto the cavity mode) should be larger than the ground state permanent dipole at the ground state equilibrium configuration.

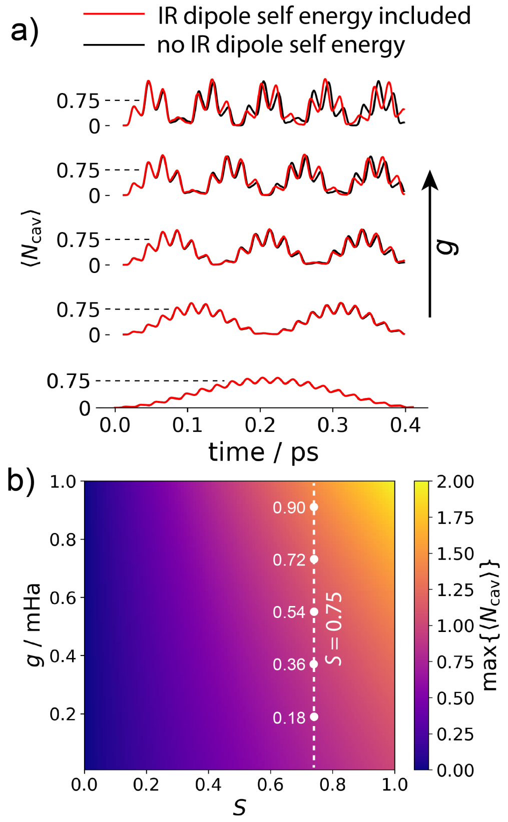

Supplementary Figure 1: (a) Time evolution of the cavity photon occupancy, $\langle \hat{N}_{\mathrm{cav}} \rangle$, for a vibration coupled to a cavity following an electronic excitation of the molecule with (red) and without (black) inclusion of the infrared (IR) dipole self energy. $\langle \hat{N}_{\mathrm{cav}} \rangle$ traces are shown for the different values of g given by the white points in (b) and for a fixed Huang-Rhys factor (S) of 0.75. (b) The maximum value of the $\langle \hat{N}_{\mathrm{cav}} \rangle$ time trace (Max$\{\langle \hat{N}_{\mathrm{cav}} \rangle\}$) as a function of both the coupling factor (g) and S, calculated including the IR dipole self energy.

Supplementary Note 4. Effect of the IR Dipole Self Energy

The term $\hat{H}_{\mathrm{DR}}$ of the total Hamiltonian (Eq. S5) given by Eq. S12 is a result of the IR dipole self energy. This term effectively alters the vibrational frequency as $\omega_v' = \sqrt{\omega_v^2 + (\omega_c \boldsymbol{A} \cdot \boldsymbol{\mu}_{\boldsymbol{\alpha}}')^2} = \sqrt{\omega_v^2 + (G_\alpha/\omega_c)^2}$ (see Eq. S15). In Figure 1a, we show a comparison of the $\langle N_{\mathrm{cav}} \rangle$ time traces from Figure 3a of the main text with and without the IR dipole self energy term included. It can be seen that only for the trace with the largest coupling value does the IR dipole self energy make a noticeable difference. Even then, the maximum $\langle N_{\mathrm{cav}} \rangle$ value does not differ significantly between with and without the IR dipole self energy. Figure 1b shows Figure 3b of the main text but with the IR dipole self energy included in the calculation.

Supplementary Note 5. Isolated Cavity-Vibration System at Non-resonant Conditions

Here we show, for an isolated cavity-vibration system, how the number of cavity photons depends on the frequency of the cavity mode, following a UV/vis excitation of the cavity coupled molecule. This calculation was done using the approximate form of the Hamiltonian, $\hat{H} = \hat{H}_0 + \hat{H}_{\text{el-vib}} + \hat{H}_{\text{cav-vib}}$.

Figure 2 is similar to Figure 3 of the main text. However, it shows how $\langle \hat{N}_{\mathrm{cav}} \rangle$ depends on the cavity mode frequency, expressed in wavenumbers, and the cavity-vibration coupling strength, g. Figure 2a shows the time evolution of $\langle \hat{N}_{\mathrm{cav}} \rangle$ following and UV/vis excitation for multiple values of ω_c and a fixed value of $g = 0.6$ mHa. Figure 2b shows the heat map of the maximum value $\langle \hat{N}_{\mathrm{cav}} \rangle$ can have ($\mathrm{Max}\{\langle \hat{N}_{\mathrm{cav}} \rangle\}$) as a function of both ω_c and g. Figure 2b indicates that as g increases, the cavity-vibration detuning can increase while still obtaining a significant $\mathrm{Max}\{\langle \hat{N}_{\mathrm{cav}} \rangle\}$ value.

The area of Figure 2 showing $\max\{\hat{N}_{\mathrm{cav}}(t)\} > 2$ can be explained by considering the

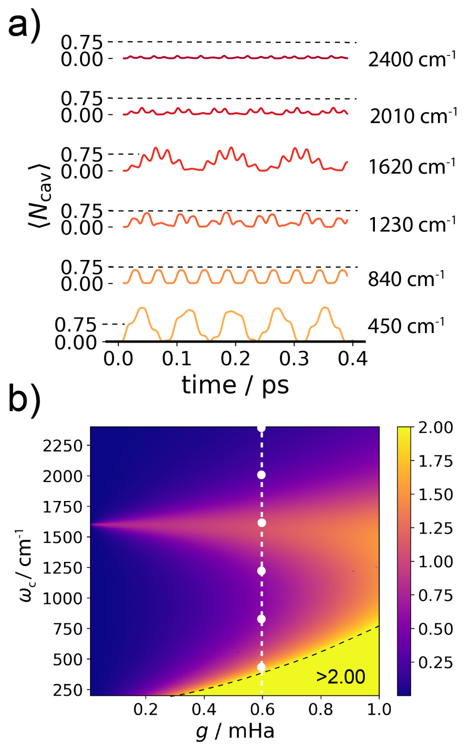

Supplementary Figure 2: (a) Time evolution of the cavity photon occupancy, ($\langle \hat{N}_{\text{cav}} \rangle$) for a single intramolecular vibration coupled to a cavity following an electronic excitation of the molecule, for different values of the cavity frequency (ω_c) in wavenumbers and for a fixed Huang-Rhys factor (S) of 0.75 and a vibrational frequency (ω_v) of 1600 cm^{-1}. (b) The maximum value of the $\langle \hat{N}_{\text{cav}} \rangle$ time trace (Max$\{\langle \hat{N}_{\text{cav}} \rangle\}$) as a function of both ω_c and the coupling strength (g). The white dashed line represents $g = 0.6$, with each point on the line corresponding to the ω_c and g values used for the traces in (a).

vibrational reorganization energy of the different polariton modes. The PES of each mode is obtained from Eq. S22 as

$$V_\gamma(\hat{R}_\gamma) = E_{\text{vert}} + \frac{\Omega_\gamma^2 \hat{R}_\gamma^2}{2} - \omega_v^2 Q_0^{(e)} c_v^{(\gamma)} \hat{R}_\gamma, \tag{S36}$$

with a minimum at

$$R_{\text{eq}}^{(\gamma)} = \frac{\omega_v^2}{\Omega_\gamma^2} c_v^{(\gamma)} Q_0^{(e)}. \tag{S37}$$

Note that $R_{\text{eq}}^{(\gamma)} = R_0$ only when $\Omega_\gamma = \omega_v$, i.e., when the vibration-cavity coupling is small, with $R_0 = Q_0^{(e)} c_v^{(\gamma)}$ being the projection of the vibrational PES displacement onto the γ

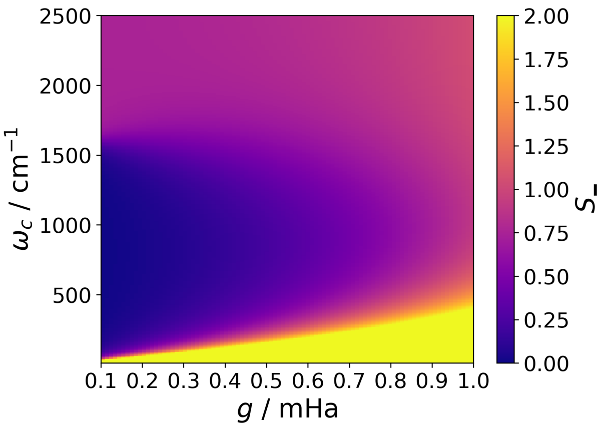

Supplementary Figure 3: The lower polariton occupation value (S_-) as a function of the cavity frequency (ω_c) and the coupling strength (g) using a fixed value of the vibrational frequency (ω_v) of 1600 cm^{-1} and Huang-Rhys factor (S) of 0.75.

polariton mode. The reorganization energy along the polariton modes is then

$$\begin{aligned}\lambda_\gamma = V_\gamma(0) - V_\gamma(R_{\text{eq}}^{(\gamma)}) &= \frac{1}{2}\frac{\omega_v^4}{\Omega_\gamma^2}(c_v^{(\gamma)}Q_0^{(e)})^2 \\ &= \frac{\omega_v^3}{\Omega_\gamma^2}\hbar S|c_v^{(\gamma)}|^2,\end{aligned} \tag{S38}$$

using $Q_0^{(e)} = \sqrt{2S\hbar/\omega_v}$. The expected polariton occupation value for the γ mode is

$$S_\gamma = \frac{\omega_v^3}{\Omega_\gamma^3}|c_v^{(\gamma)}|^2 S, \tag{S39}$$

where $S_\gamma = \lambda_\gamma/\hbar\Omega_\gamma$ was used. Eq. S39 gives the maximum number of polaritons that can be converted into cavity photons but does not account for interference effects of the two polariton oscillating wave packets projected onto the cavity axis. As the cavity is red-detuned from the vibrational mode, the lower polariton axis becomes close to the cavity axis (i.e., the lower polariton has a large cavity character) but due to the large coupling values studied, the lower polariton retains some vibration character and its equilibrium position remains non-zero unlike the minimum of the bare cavity PES. As the cavity is red-detuned the number of cavity photons is determined largely by the projection of the lower polariton wavepacket onto the cavity axis and S_- becomes a good measure of $\max\{\hat{N}_{\text{cav}}(t)\}$. A heat

map of S_- as a function of g and ω_c is presented in Figure 3 showing good agreement with Figure 2 at low ω_c values. $\max\{\hat{N}_{\text{cav}}(t)\}$ being large at small ω_c values is the result of λ_- being converted into many cavity photons due to their small photon frequency as well as the strong coupling causing the lower polariton mode to retain vibrational character. Essentially, at large g values, Ω_γ decreases more quickly with ω_c than $c_v^{(\gamma)}$ once ω_c becomes small enough, leading to S_- becoming very large. This effect could potentially be used to maximize the photonic down-conversion process if large g values are obtainable experimentally.

Supplementary Note 6. Dissipative Cavity-Vibration System at Non-resonant Conditions

Here, multiple % emission heat maps of the ESVP mediated photonic down conversion process are shown for different cavity mode frequencies (ω_c). Each heat map shows % emission as a function of g and $\mathcal{Q}^{1/2}$, while keeping the frequency of the vibrational mode fixed at $\omega_v = 1600$ cm^{-1}.

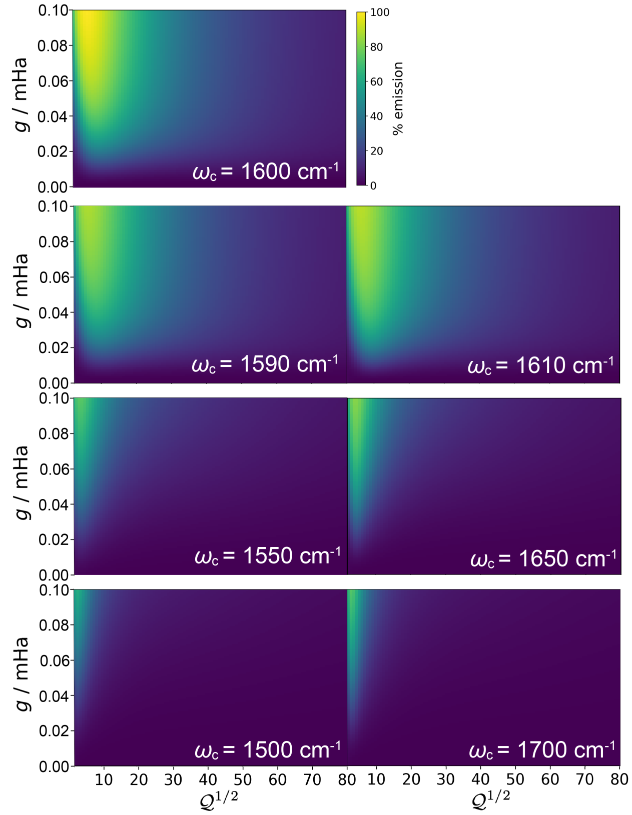

Supplementary Figure 4: Heat maps of the % emission of infrared (IR) photons produced by the excited state vibrational polariton (ESVP) mediated photonic down conversion as a function of the coupling constant (g) and the square root of the quality factor ($\mathcal{Q}$), for different frequencies of the cavity mode (ω_c).

Supplementary Note 7. Effect of the Non-Radiative Lifetime on % Emission for the Open Cavity-Vibration System

Here, similar to section , multiple % emission heat maps of the ESVP mediated photonic down conversion process are shown, but for different vibrational decay lifetimes. The vibrational decay lifetime is defined as the lifetime of the vibration when uncoupled to the cavity but coupled to a bath of harmonic oscillators representing non-radiative dissipation. The vibrational decay lifetime (τ) is related to the vibrational damping constant, η_B, by $\eta_B = 1/2\pi\omega_c\tau$.

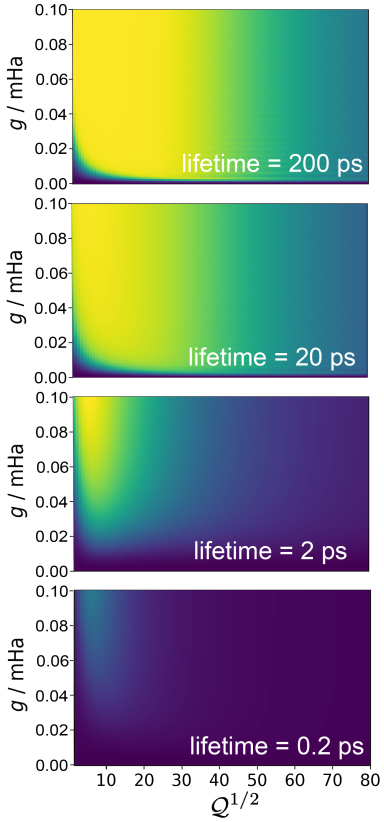

Supplementary Figure 5: Heat maps of the % emission of infrared (IR) photons produced by the excited state vibrational polaritons (ESVP) mediated photonic down conversion as a function of the coupling strength (g) and square root of the quality factor ($\mathcal{Q}$), for different vibrational decay lifetimes.

Supplementary Note 8. Absorption Response Function for Pyrene

The Figure below shows the calculated response function of pyrene as a clarifying example of how the absorption spectra were calculated in this work. The large number of vibrational modes of differing frequencies within pyrene, along with the inhomogenoeus broadening of the electronic transition (σ_{E_0}) causes the response function to go to zero at longer times. For a more detailed description of the absorption calculations, see Provazza et. al.[2]

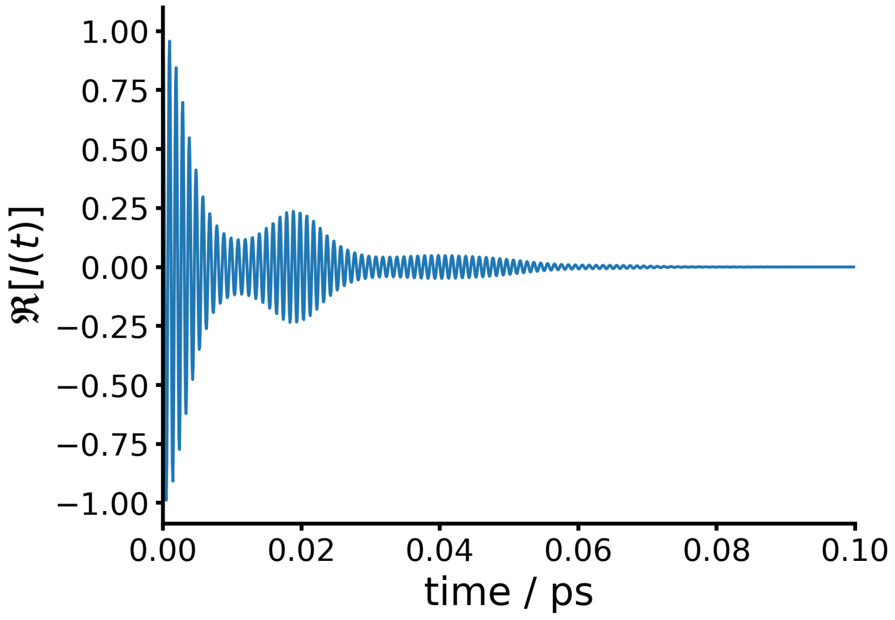

Supplementary Figure 6: Real component of the response function ($\Re[I(t)]$) for the linear absorption of pyrene calculated by Eq. (44) in the main text. Temperature (T) was set at $T = 300$ K and inhomogeneous broadening (σ_{E_0}) at 200 cm^{-1}. The Fourier transform of $I(t)$ shown here produces the calculated absorption spectrum of pyrene used in Figure 5a of the main text.

Supplementary references

(1) Mandal, A.; Montillo Vega, S.; Huo, P. Polarized Fock States and the Dynamical Casimir Effect in Molecular Cavity Quantum Electrodynamics. Journal of Physical Chemistry

Letters **2020**, 11, 9215–9223.

(2) Provazza, J.; Tempelaar, R.; Coker, D. F. Analytic and numerical vibronic spectra from quasi-classical trajectory ensembles. Journal of Chemical Physics **2021**, 155.
